# A Lightweight Three-Factor Authentication and Key Agreement Scheme in Wireless Sensor Networks for Smart Homes

**DOI:** 10.3390/s19092012

**Published:** 2019-04-29

**Authors:** Sooyeon Shin, Taekyoung Kwon

**Affiliations:** Graduate School of Information, Yonsei University, Seoul 03722, Korea; shinsy80@yonsei.ac.kr

**Keywords:** three-factor authentication, key agreement, password, smart card, biometrics, anonymity, untraceability, wireless sensor networks, Internet of Things, smart home

## Abstract

A wireless sensor network (WSN) is used for a smart home system’s backbone that monitors home environment and controls smart home devices to manage lighting, heating, security and surveillance. However, despite its convenience and potential benefits, there are concerns about various security threats that may infringe on privacy and threaten our home life. For protecting WSNs for smart homes from those threats, authentication and key agreement are basic security requirements. There have been a large number of proposed authentication and key agreement scheme for WSNs. In 2017, Jung et al. proposed an efficient and security enhanced anonymous authentication with key agreement scheme by employing biometrics information as the third authentication factor. They claimed that their scheme resists on various security attacks and satisfies basic security requirements. However, we have discovered that Jung et al.’s scheme possesses some security weaknesses. Their scheme cannot guarantee security of the secret key of gateway node and security of session key and protection against user tracking attack, information leakage attack, and user impersonation attack. In this paper, we describe how those security weaknesses occur and propose a lightweight three-factor authentication and key agreement scheme in WSNs for smart homes, as an improved version of Jung et al.’s scheme. We then present a detailed analysis of the security and performance of the proposed scheme and compare the analysis results with other related schemes.

## 1. Introduction

Wireless sensor networks (WSNs), composed of many low-cost and low-power sensor nodes, have become a popular technology for various applications including Internet of Things (IoT) applications such as health-care, smart homes, smart factoring, and smart city [1]. For example, a smart home is defined as a networking technology to integrate devices and appliances so that many smart home devices with sensors monitor home environments, capture users’ offline activities, and control lighting, windows, doors, heating, security and surveillance, and so on. In the smart home application, internal and external users need to directly access the WSN for real-time control and data acquisition will increase through direct access. According to Zion Market Research [2], the global smart home market was valued at USD 39.68 billion in 2017 and is expected to reach a value of USD 159.68 billion by 2023. The growing market for smart home provides a more comfortable and easier way of life to users while presenting new challenges for preserving privacy. Moreover, due to the inherent characteristics of WSNs, such as resource constraints and the use of wireless medium, they are likely to be exposed to various attacks. In such situations, cryptographic techniques such as encryption and message authentication should be applied to protect user privacy and WSN against various attacks. To apply cryptographic techniques, user authentication and key agreement are basically required.

### 1.1. Related Works

To improve security of WSNs, many user authentication and key agreement schemes have been proposed in the last decade [3,4,5,6,7,8,9,10,11,12,13,14]. In 2006, based on lightweight operations, such as XOR operations and one-way hash function, Wong et al. [3] proposed a lightweight strong-password authentication scheme for WSNs. However, Das [4] pointed out that Wong et al.’s scheme is vulnerable to same login identity attack, replay attack and stolen-verifier attack. Das then presented a two-factor authenticated key establishment scheme for WSNs as an improved version of Wong et al.’s scheme. Unfortunately, many papers [5,15,16,17,18] have revealed that Das’s scheme is vulnerable to various attacks such as privileged insider, gateway node bypass, smart card loss, and parallel session attacks.

Although many improved versions of Das’s scheme have been proposed to solve the above-mentioned security flaws, they still have some security problems. As one of the improved versions, Vaidya et al. [5] proposed a novel two-factor user authentication scheme with key agreement for WSNs, but in 2014, Kim et al. [6] pointed out that Vaidya et al.’s scheme could not withstand both user impersonation attack and gateway node bypass attack. Kim et al. then proposed a user authentication and key agreement scheme that resisted those attacks. In 2015, Chang et al. [8] found that Kim et al.’s scheme is vulnerable to impersonation, lost smart card, man-in-the-middle attacks and does not provide session key security and user privacy. Chang et al. then presented an enhanced two-factor authentication and key agreement using dynamic identities. However, recently, Park et al. [9] and Jung et al. [13] pointed out that Chang et al.’s scheme has security flaws such as off-line password guessing attack, user impersonation attack, perfect forward secrecy problem, and incorrectness of password change. Park et al. and Jung et al. then proposed improved schemes in 2016 and 2017, respectively. Park et al. proposed a three-factor user authentication and key agreement scheme using ECC (Elliptic Curve Cryptosystem) and Jung et al. proposed an efficient anonymous authentication with key agreement scheme using only lightweight operations. However, we found that Jung et al.’s scheme still has some security weaknesses [19].

On the other hand, user authentication and key agreement schemes based on the concept of IoT have been proposed. In 2014, Turkanović et al. [7] proposed an energy-efficient user authentication scheme with high security and low computational cost using the concept of the IoT. However, Farash et al. [10] found that Turkanović et al.’s scheme has security weaknesses and then proposed an improved scheme. In 2016, Amin et al. [11] claimed that Farash et al.’s scheme has some security problems such as known session-specific temporary information attack, off-line password guessing attack using a stolen-smart card, a new-smart card-issue attack, user impersonation attack, insecurity of the secret key of the gateway node, and insecurity of user anonymity. Amin et al. then proposed an anonymity-preserving three-factor authenticated key exchange scheme for IoT-based WSNs. Unfortunately, recently, Jiang et al. [12] found several security flaws in Amin et al.’s scheme, such as smart card loss attack, known session-specific temporary information attack, and tracking attack. Jiang et al. then proposed a lightweight three-factor authentication and key agreement scheme for Internet-integrated WSNs based on Rabin cryptosystem. Jiang et al.’s scheme provides various security features but this scheme is difficult to implement and deploy in practical applications because of heavyweight decryption of Rabin cryptosystem.

### 1.2. Research Contributions

As shown in the section on related works, most of the proposed user authentication and key agreement schemes for WSNs fail to provide adequate security protection or still suffer from various security attacks. To overcome these weaknesses, we design a lightweight authentication and key agreement scheme. Our research contributions are as follows.

- We analyze the most recent three-factor authentication and key agreement scheme of Jung et al.’s scheme and present its security weaknesses. We show that Jung et al.’s scheme [13] does not provide strong anonymity and the secrecy of the secret key of the gateway node. We also show that Jung et al.’s scheme is vulnerable to a tracing attack, information leakage attack, session key recovery attack, and user impersonation attack.
- We introduce a system model suitable for smart homes based on WSNs. Under this model, we propose a lightweight three-factor authentication and key agreement scheme as an improved version of Jung et al.’s scheme. The proposed scheme not only satisfies various security requirements but also uses lightweight operations, such as XOR and hash functions, which are very suitable for the resource constrained WSNs.
- We formally prove the security of the proposed scheme using both random oracle model and BAN (Burrows-Abadi-Needham) logic. We then verify the proposed scheme on popular and robust security verification tool, AVISPA (Automated Validation of Internet Security Protocols and Applications).
- Through informal security analysis, we show that the proposed scheme can satisfy the required security properties and withstand various attacks. We then compare it with other related schemes in terms of security features.
- Through a performance evaluation, we compare the performance of the proposed scheme with other related schemes in terms of their computational cost and communication cost.

The remaining parts of this paper are as follows: Section 2 briefly reviews Jung et al.’s scheme; Section 3 demonstrates the security weaknesses of Jung et al.’s scheme; the details of the proposed scheme is illustrated in Section 4; Section 5 and Section 6 give the formal and informal security analysis of the proposed scheme, respectively; Section 7 shows the performance evaluation of the proposed scheme; Section 8 concludes the paper.

### 1.3. Preliminary

A conventional hash function may return different outputs even if there is little variation in the inputs because its output is sensitive [20]. On the other hand, since biometric information is prone to various noises during data acquisition, it is difficult to re-product actual biometric in common practice. A fuzzy extractor method has been used to solve these problems [20,21,22,23]. The fuzzy extractor can extract a uniformly-random string and a public information from the biometric template with a given error tolerance *t*. In other words, even if the input changes slightly, the fuzzy extractor could output the same random string with the help of the public information. The fuzzy extractor consists of the following two algorithms.

- $GEN\left(Bio_{i}\right)=\left(b_{i},par_{i}\right)$: Given a biometric template $Bio_{i}$ as an input, this probabilistic algorithm outputs a secret biometric key $b_{i}$ and a helper string $par_{i}$.
- $REP\left(Bio_{i}^{\prime },par_{i}\right)=\left(b_{i}\right)$: Given a noisy biometric $Bio_{i}^{\prime }$ and a helper string $par_{i}$ as inputs, this deterministic algorithm reproduces the biometric key $b_{i}$.

## 2. Review of Jung et al.’s Scheme

In this section, we briefly review Jung et al.’s anonymous authentication with key agreement scheme in WSNs [13]. Jung et al.’s scheme consists of four phases: user registration, login, authentication, and password change. We describe the first three phases related to the security weaknesses in detail. Table 1 shows the notations used in Jung et al.’s scheme.

Before a sensor node $S_{j}$ is deployed, it keeps $SID_{j}$ and $X_{S_{j}}^{*}$ in its storage, where $X_{S_{j}}^{*}=h(SID_{j}\left||K\right)$.

### 2.1. Registration Phase

In the registration phase, $U_{i}$ sends a request message for registration to GWN then GWN issues a smart card for $U_{i}$. All messages in this phase are transmitted through a secure channel.

(1) $U_{i}$ chooses $ID_{i},PW_{i}$, and a random number *u* and imprints his/her biometrics $Bio_{i}$. $U_{i}$ computes $HPW_{i}=h(PW_{i}||H\left(Bio_{i}\right))$ and $TID_{i}=h(ID_{i}\left||u\right)$ and sends a registration request $\langle TID_{i},HPW_{i}\rangle$ to GWN.
(2) Upon receiving the registration request, GWN computes $HID_{i}=h(TID_{i}\left||K\right)⊕HPW_{i},A_{i}=h(HPW_{i}||TID_{i})⊕HID_{i},B_{i}=h(HPW_{i}||HID_{i}),$ and $C_{i}=HID_{i}⊕K$. GWN then issues a smart card by storing $\left(A_{i},B_{i},C_{i},h\left(\cdot \right),H\left(\cdot \right)\right)$ in its memory and sends the smart card to $U_{i}$.
(3) Upon receiving the smart card, $U_{i}$ computes $D_{i}=u⊕H\left(Bio_{i}\right)$ and additionally stores it into the smart card.

### 2.2. Login Phase

In the login phase, $U_{i}$ sends the service request to GWN using his/her smart card, identity, password, and biometric information.

(1) $U_{i}$ inserts own smart card into a terminal, enters $ID_{i}$ and $PW_{i}$, and imprints $Bio_{i}$.
(2) The smart card computes $HPW_{i}^{*}=h(PW_{i}||H\left(Bio_{i}\right)),$$u=D_{i}⊕H\left(Bio_{i}\right),TID_{i}=h(ID_{i}||u),HID_{i}^{*}=A_{i}⊕h(HPW_{i}^{*}||TID_{i})$, and $B_{i}^{*}=h(HPW_{i}^{*}||HID_{i}^{*})$. The smart card then checks whether $B_{i}^{*}$ matches with the received $B_{i}$. If it does not hold, the smart card terminates this phase. Otherwise, the smart card confirms the legitimacy of $U_{i}$ and computes $DID_{i}=TID_{i}⊕HID_{i}^{*}$ and $M_{U_{i},G}=h(TID_{i}||HPW_{i}^{*}||HID_{i}^{*}\left|\right|T_{1})$.
(3) The smart card sends the login request $\langle DID_{i},M_{U_{i},G},$$C_{i},T_{1}\rangle$ to GWN through a public channel.

### 2.3. Authentication Phase

The authentication phase begins when GWN receives the login request from $U_{i}$. In this phase, $U_{i},GWN$, and $S_{j}$ authenticate each other and establish a session key $K_{S}$.

(1) GWN checks the validity of $T_{1}$ and computes $TID_{i}^{*}=DID_{i}⊕C_{i}⊕K,HID_{i}=C_{i}⊕K,HPW_{i}^{*}=HID_{i}⊕h(TID_{i}\left||K\right)$ and $M_{U_{i},G}^{*}=h(TID_{i}^{*}||HPW_{i}^{*}||HID_{i}\left|\right|T_{1})$. GWN then checks whether $M_{U_{i},G}^{*}$ matches with the received $M_{U_{i},G}$. If it does not hold, it terminates this phase. Otherwise, GWN believes that $U_{i}$ is authentic and proceeds with the next step.
(2) GWN chooses a random number *R* and computes $X_{S_{j}}=h(SID_{j}||K),M_{j}=R⊕X_{S_{j}},K_{S}=f\left(DID_{i},R\right)$ and $M_{G,S_{j}}=h(DID_{i}||SID_{j}\left|\right|X_{S_{j}}\left|\right|K_{S}\left|\right|$$T_{2})$. GWN then sends the message $\langle DID_{i},M_{G,S_{j}},$$M_{j},T_{2}\rangle$ to $S_{j}$ through a public channel.
(3) Upon receiving the message, $S_{j}$ checks the validity of $T_{2}$ and computes $R^{*}=M_{j}⊕X_{S_{j}}^{*},K_{S}^{*}=f\left(DID_{i},R^{*}\right),$ and $M_{G,S_{j}}^{*}=h(DID_{i}||SID_{j}\left|\right|X_{S_{j}}^{*}\left|\right|K_{S}^{*}\left|\right|T_{2})$. $S_{j}$ checks whether $M_{G,S_{j}}^{*}$ matches with the received $M_{G,S_{j}}$. If it does not hold, $S_{j}$ terminates this phase. Otherwise, $S_{j}$ believes the GWN is authentic.
(4) $S_{j}$ computes $k_{j}=h(X_{S_{j}}^{*}\left|\right|T_{3})$ and $M_{S_{j},G}=h(k_{j}\left|\right|X_{S_{j}}^{*}\left|\right|K_{S}^{*}\left|\right|T_{3})$. $S_{j}$ finally sends the message $\langle M_{S_{j},G},T_{3}\rangle$ to GWN through a public channel.
(5) Upon receiving the message, GWN checks the validity of $T_{3}$ and computes $k_{j}=h(X_{S_{j}}\left|\right|T_{3})$ and $M_{S_{j},G}^{*}=h(k_{j}\left|\right|X_{S_{j}}\left|\right|K_{S}\left|\right|T_{3})$. GWN then checks whether $M_{S_{j},G}^{*}$ matches with the received $M_{S_{j},G}$. If it does not hold, GWN terminates this phase. Otherwise, GWN believes that $S_{j}$ is authentic and proceeds with the next step.
(6) GWN computes $k_{i}=R⊕h(TID_{i}^{*}\left||K\right)$ and $M_{G,U_{i}}=h(K_{S}\left|\right|k_{i}\left|\right|T_{4})$ and sends the message $\langle k_{i},M_{G,U_{i}},T_{4}\rangle$ to $U_{i}$ through a public channel.
(7) Upon receiving the message, $U_{i}$ checks the validity of $T_{4}$ and computes $R^{*}=k_{i}⊕HPW_{i}⊕HID_{i}^{*},K_{S}^{*}=f\left(DID_{i},R^{*}\right),$ and $M_{G,U_{i}}^{*}=h\left(K_{S}^{*},k_{i},T_{4}\right)$. $U_{i}$ then checks whether $M_{G,U_{i}}^{*}$ matches with the received $M_{G,U_{i}}$. If it does not hold, this phase is terminated. Otherwise, $U_{i}$ believes that GWN is authentic and successfully ends the authentication phase.

## 3. Security Weaknesses of Jung et al.’s Scheme

In this section, we show that Jung et al.’s scheme [13] has security weaknesses.

### 3.1. Tracing Attack

As the concern for privacy increases in our lives, user anonymity has become a vital security requirement in various applications including WSN applications. For example, the personalized services in smart home applications (e.g., home energy management system) provide users with better convenience, but breach of privacy has been a serious concern [24]. In general, the preservation of identity privacy in the context of an authentication protocol requires not only anonymity but also untraceability [25]. Although untraceability is not a necessary condition of anonymity, strong anonymity with untraceability is required for fully protecting user privacy. In Jung et al.’s scheme, every time $U_{i}$ uses the fixed values $DID_{i}$ and $C_{i}$ to login the WSN thus anyone can trace $U_{i}$ according to these strings constantly. Therefore, Jung et al.’s scheme is prone to user tracing attack and fails to provide untraceability.

### 3.2. Insecurity of the Secret Key of the Gateway Node

In Jung et al.’s scheme, the secret key *K* of GWN is used to compute critical parameters of users’ smart cards and secret keys of all sensor nodes. The security of Jung et al.’s scheme thus depends on the security of the secret key *K*. Unfortunately, any authorized user can easily extract *K* using his/her identity, password, biometrics and values stored in the smart card. Assume that an authorized user $U_{i}$ retrieves the information $\langle A_{i},B_{i},C_{i},D_{i}\rangle$ from his/her smart card, where $A_{i}=h(HPW_{i}||TID_{i})⊕HID_{i}$, $B_{i}=h(HPW_{i}||HID_{i})$, $C_{i}=HID_{i}⊕K$, and $D_{i}=u⊕H\left(Bio_{i}\right)$. As the smart card calculates at the login phase, $U_{i}$ then computes $u=D_{i}⊕H\left(Bio_{i}\right)$, $TID_{i}^{\prime }=h(ID_{i}\left||u\right)$, and $HID_{i}^{\prime }=A_{i}⊕h(HPW_{i}||TID_{i}^{\prime })$. Based on $HID_{i}^{\prime }$ and $C_{i}$, $U_{i}$ computes $K^{\prime }$, where $K^{\prime }=C_{i}⊕HID_{i}^{\prime }$. Since he or she now knows the secret key $K^{\prime }$, $U_{i}$ can impersonate GWN and launch the following attacks.

### 3.3. Information Leakage Attack

We described how an authorized user $U_{j}$ can know $K^{\prime }$ in Section 3.2. After getting $K^{\prime }$, $U_{j}$ who acts as an adversary A can achieve secret information required for authentication and key agreement as follows:

(1) A intercepts the user $U_{i}$’s login message $\langle DID_{i},M_{U_{i},G},$$C_{i},T_{1}\rangle$, where $DID_{i}=TID_{i}⊕HID_{i}$, $M_{U_{i},G}=h(TID_{i}||HPW_{i}||HID_{i}\left|\right|T_{1})$, and $C_{i}=HID_{i}⊕K$.
(2) A computes $HID_{i}^{\prime }=C_{i}⊕K^{\prime }$ and $TID_{i}^{\prime }=DID_{i}⊕HID_{i}^{\prime }$.
(3) A then computes $HPW_{i}^{\prime }=HID_{i}^{\prime }⊕h(TID_{i}^{\prime }\left|\right|K^{\prime })$.

Thus, A can obtain all secret values $TID_{i}^{\prime },HID_{i}^{\prime },$ and $HPW_{i}^{\prime }$ need to login the WSN and launch session key recovery attack and user impersonation attack.

### 3.4. Session Key Compromise

We assume that A can obtain the secret information by intercepting the $U_{i}$’s login message and also can intercept the last message of the authentication phase. After getting the secret information in Section 3.3 and $k_{i}$, A can successfully launch a session key recovery attack as follows:

(1) A intercepts the last message $\langle k_{i},M_{G,U_{i}},T_{4}\rangle$ sent from GWN, where $k_{i}=R⊕h(TID_{i}\left|\right|K^{\prime })$ and $M_{G,U_{i}}=h(K_{S}\left|\right|k_{i}\left|\right|T_{4})$.
(2) A computes $R^{\prime }=k_{i}⊕h(TID_{i}^{\prime }\left||K\right)$.
(3) A discovers the session key $K_{S}^{\prime }$ between the user $U_{i}$, GWN, and the sensor node $S_{j}$ by computing $K_{S}^{\prime }=f(DID_{i}\left|\right|R^{\prime })$.

Thus, according to the above procedure, an adversary can successfully construct the session key $K_{S}^{\prime }$ between $U_{i}$, GWN, and $S_{j}$.

### 3.5. User Impersonation Attack

Once an adversary A achieves the GWN’s secret key *K* and secret information $TID_{i}^{\prime },HID_{i}^{\prime }$, and $HPW_{i}^{\prime }$ as described in Section 3.2 and Section 3.3, respectively, A can also impersonate a user $U_{i}$ in Jung et al.’s scheme without the target user’s identity $ID_{i}$, password $PW_{i}$, and biometric information $Bio_{i}$ as follows:

(1) A computes $DID_{i}^{\prime }=TID_{i}^{\prime }⊕HID_{i}^{\prime },C_{i}^{\prime }=HID_{i}^{\prime }⊕K^{\prime }$ and $M_{U_{i},G}^{\prime }=h(TID_{i}^{\prime }||HPW_{i}^{\prime }||HID_{i}^{\prime }\left|\right|T_{1}^{\prime })$, where $T_{1}^{\prime }$ is the current time stamp used by A. Of course, since $DID_{i}$ and $C_{i}$ are the fixed values, it is possible to use the previously intercepted one.
(2) A sends the login message $\langle DID_{i}^{\prime },M_{U_{i},G}^{\prime },C_{i}^{\prime },T_{1}^{\prime }\rangle$.
(3) At GWN, user authentication is successfully performed and A calculates the session key $K_{S}^{\prime }$ after receiving the last message as described in Section 3.4.

It is clear from the above discussion that A can masquerade as a valid user $U_{i}$ to login to the WSN without $ID_{i}$, $PW_{i}$ and $Bio_{i}$. Thus, Jung et al.’s scheme is vulnerable to the user impersonation attack.

## 4. Proposed Scheme for Smart Homes

**Figure 1:**
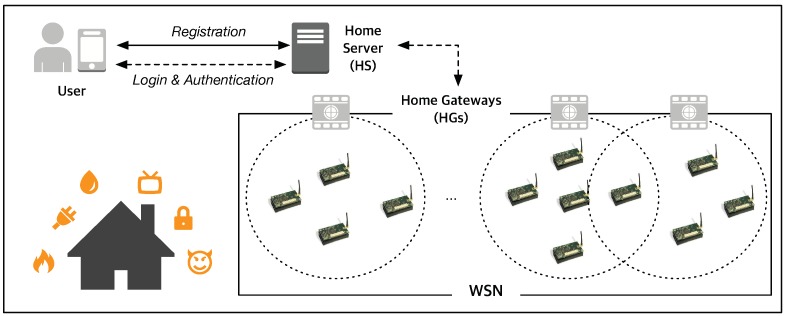
An example of smart home monitoring and control system based on WSNs.

In this section, we propose a three-factor authentication and key agreement scheme in WSNs for smart homes in which we find the aforementioned security weaknesses found in Jung et al.’s scheme. Figure 1 illustrates a system model of WSNs for a smart home monitoring and control system. The system model includes three types of entities: a user ($U_{i}$), a home gateway node (HG), and sensor nodes ($S_{j}$). After registration and mutual authentication with the help of HG, $U_{i}$ can access the WSN to monitor and control smart home.

The proposed scheme consists of five phases: system setup, user registration, login, authentication, and password change. We use the additional notation for the proposed scheme listed in Table 2.

### 4.1. System Setup Phase

This phase is executed by home gateway (HG) in an off-line mode before deployment of sensor nodes in a target field.

(1) HG generates randomly two master secrets $K_{U}$ and $K_{S}$ for all users and all sensor nodes, respectively, which are only known to HG.
(2) HG selects a unique identity $SID_{j}$ and computes $X_{S_{j}}=h(SID_{j}\left|\right|K_{S})$ for each sensor node $S_{j}$.
(3) Finally, each sensor node is deployed in the target field after storing $SID_{j}$ and $X_{S_{j}}$ into its memory in a secure manner.

### 4.2. User Registration Phase

**Figure 2:**
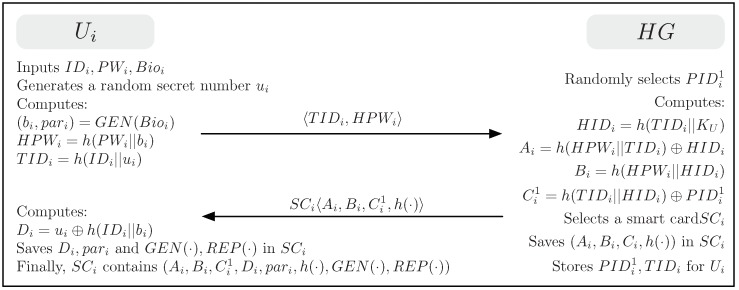
User registration phase for the proposed scheme.

The user registration phase begins when a user $U_{i}$ sends a request message for registration to HG over a secure channel. Figure 2 illustrates the user registration phase for the proposed scheme. This phase is described below.

(1) $U_{i}$ selects the desired identity $ID_{i}$ and password $PW_{i}$ and imprints his/her biometrics $Bio_{i}$. $U_{i}$ generates a random secret number $u_{i}$ and computes $\left(b_{i},par_{i}\right)=GEN\left(Bio_{i}\right)$, $HPW_{i}=h(PW_{i}\left|\right|b_{i})$ and $TID_{i}=h(ID_{i}\left|\right|u_{i})$. $U_{i}$ then sends a registration request $\langle TID_{i},HPW_{i}\rangle$ to HG over a secure channel.
(2) Upon receiving the user’s registration request, HG randomly selects a unique one-time pseudonym $PID_{i}^{1}$ for $U_{i}$. HG computes $HID_{i}=h(TID_{i}\left|\right|K_{U})$, $A_{i}=h(HPW_{i}||TID_{i})⊕HID_{i}$, $B_{i}=h(HPW_{i}||HID_{i})$, and $C_{i}^{1}=h(TID_{i}||HID_{i})⊕PID_{i}^{1}$. HG issues a smart card $SC_{i}$ for $U_{i}$ after saving $\left\{A_{i},B_{i},C_{i}^{1},h\left(\cdot \right)\right\}$ in it. HG then sends $SC_{i}$ to $U_{i}$ over a secure channel and stores $\left\{PID_{i}^{1},TID_{i}\right\}$ into its memory.
(3) After receiving the smart card $SC_{i}$, $U_{i}$ computes $D_{i}=u⊕h(ID_{i}\left|\right|b_{i})$ and saves $D_{i}$, $par_{i}$, GEN(·), and REP(·) in $SC_{i}$. Finally, $SC_{i}$ contains $\left\{A_{i},B_{i},C_{i}^{1},D_{i},par_{i},h\left(\cdot \right),GEN\left(\cdot \right),REP\left(\cdot \right)\right\}$.

### 4.3. Login Phase

**Figure 3:**
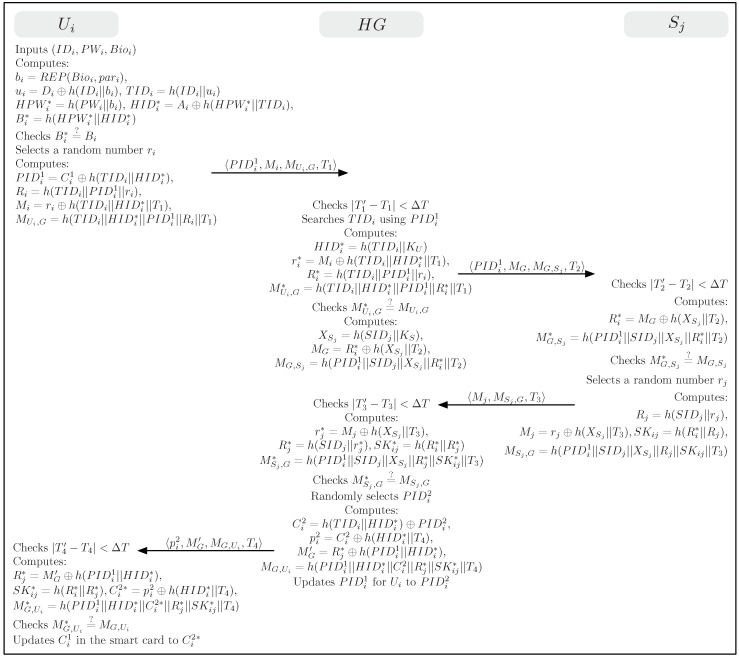
Login and authentication phases for the proposed scheme.

The login phase is executed when $U_{i}$ wants to gain access to the WSN using his/her $SC_{i},ID_{i},PW_{i}$, and $Bio_{i}$. Figure 3 illustrates the login and authentication phases for the proposed scheme. This phase contains the following steps.

(1) $U_{i}$ inserts own $SC_{i}$, inputs his/her $ID_{i}$ and $PW_{i}$, and imprints his/her biometrics $Bio_{i}$ into a terminal (i.e., a smart card reader or a smartphone embedded with $SC_{i}$).
(2) $SC_{i}$ computes $b_{i}=REP\left(Bio_{i},\beta_{i}\right)$, $u_{i}=D_{i}⊕h(ID_{i}\left|\right|b_{i})$, $TID_{i}=h(ID_{i}\left|\right|u_{i})$, $HID_{i}^{*}=A_{i}⊕h(HPW_{i}^{*}||TID_{i})$, and $B_{i}^{*}=h(HPW_{i}^{*}||HID_{i}^{*})$. $SC_{i}$ checks whether $B_{i}^{*}$ matches with the stored $B_{i}$. If it matches $SC_{i}$ ensures that $U_{i}$ has provided correct $ID_{i},PW_{i},$ and $Bio_{i}$. $SC_{i}$ then selects a random number $r_{i}$ and computes $PID_{i}^{1}=C_{i}^{1}⊕h(TID_{i}||HID_{i}^{*}),R_{i}=h(TID_{i}||PID_{i}^{1}\left|\right|r_{i}),M_{i}\;=\;r_{i}⊕h(TID_{i}||HID_{i}^{*}\left|\right|T_{1})$ and $M_{U_{i},G}=h(TID_{i}||HID_{i}^{*}\left|\right|$$PID_{i}^{1}\left|\right|R_{i}\left|\right|T_{1})$.
(3) Finally, $U_{i}$ sends a login request $\langle PID_{i}^{1},M_{i},M_{U_{i},G},T_{1}\rangle$ to HG over a public channel.

### 4.4. Authentication Phase

The authentication phase begins when HG receives the login request from $U_{i}$. For achieving mutual authentication and session key agreement, this phase executes in several steps as followings.

(1) HG checks the validity of the timestamp $|T_{1}^{\prime }-T_{1}|<\Delta T$ and searches $TID_{i}$ using $PID_{i}^{1}$. HG computes $HID_{i}^{*}=h(TID_{i}\left|\right|K_{U})$, $r_{i}^{*}=M_{i}⊕h(TID_{i}||HID_{i}^{*}\left|\right|T_{1})$, $R_{i}^{*}=h(TID_{i}||PID_{i}^{1}\left|\right|r_{i}^{*})$, and $M_{U_{i},G}^{*}=h(TID_{i}||HID_{i}^{*}||PID_{i}^{1}\left|\right|R_{i}^{*}\left|\right|T_{1})$. Then, HG compares $M_{U_{i},G}^{*}$ with the received value $M_{U_{i},G}$. If this condition is not satisfied, HG terminates this phase. Otherwise, HG believes that $U_{i}$ is a legitimate user. HG then chooses an appropriate sensor node $S_{j}$ for the user’s needs and computes $X_{S_{j}}=h(SID_{j}\left|\right|K_{S})$, $M_{G}=R_{i}^{*}⊕h(X_{S_{j}}\left|\right|T_{2})$, and $M_{G,S_{j}}=h(PID_{i}^{1}||SID_{j}\left|\right|X_{S_{j}}\left|\right|R_{i}^{*}\left|\right|T_{2})$. HG sends the message $\langle PID_{i}^{1},M_{G},M_{G,S_{j}},T_{2}\rangle$ to $S_{j}$ over a public channel.
(2) Upon receiving the message from HG, $S_{j}$ checks the validity of the timestamp $|T_{2}^{\prime }-T_{2}|<\Delta T$ and compute $R_{i}^{*}=M_{G}⊕h(X_{S_{j}}\left|\right|T_{2})$ and $M_{G,S_{j}}=h(PID_{i}^{1}||SID_{j}\left|\right|X_{S_{j}}\left|\right|R_{i}^{*}\left|\right|T_{2})$. $S_{j}$ then compares $M_{G,S_{j}}^{*}$ with the received value $M_{G,S_{j}}$. If this condition is not satisfied, $S_{j}$ terminates this phase since HG fails to prove to be a legitimate home gateway. Otherwise, $S_{j}$ believes that HG is authentic. $S_{j}$ then selects a random number $r_{j}$ and computes $R_{j}=h(SID_{j}\left|\right|r_{j})$, $M_{j}=r_{j}⊕h(X_{S_{j}}\left|\right|T_{3})$, $SK_{ij}=h(R_{i}^{*}\left|\right|R_{j})$, and $M_{S_{j},M}=h(PID_{i}^{1}||SID_{j}\left|\right|X_{S_{j}}\left|\right|R_{j}||SK_{ij}\left|\right|T_{3})$. $S_{j}$ sends the message $\langle M_{j},M_{S_{j},G},T_{3}\rangle$ to HG over a public channel.
(3) Upon receiving the message from $S_{j}$, HG checks the validity of the timestamp $|T_{3}^{\prime }-T_{3}|<\Delta T$ and computes $r_{j}^{*}=M_{j}⊕h(X_{S_{j}}\left|\right|T_{3})$, $R_{j}^{*}=h(SID_{j}\left|\right|r_{j}^{*})$, $SK_{ij}^{*}=h(R_{i}^{*}\left|\right|R_{j}^{*})$, and $M_{S_{j},G}^{*}=h(PID_{i}^{1}||SID_{j}\left|\right|X_{S_{j}}\left|\right|R_{j}^{*}||SK_{ij}^{*}\left|\right|T_{3})$. HG compares $M_{S_{j},G}^{*}$ with the received value $M_{S_{j},G}$. If this condition is not satisfied, HG terminates this phase. Otherwise, HG believes that $S_{j}$ is a legitimate sensor node. HG then randomly selects another unique one-time pseudonym $PID_{i}^{2}$ for $U_{i}$’s next login session and computes $C_{i}^{2}=h(TID_{i}||HID_{i}^{*})⊕PID_{i}^{2}$, $p_{i}^{2}=C_{i}^{2}⊕h(HID_{i}^{*}\left|\right|T_{4})$, $M_{G}^{\prime }=R_{j}^{*}⊕h(PID_{i}^{1}||HID_{i}^{*})$, and $M_{G,U_{i}}=h(PID_{i}^{1}||HID_{i}^{*}\left|\right|C_{i}^{2}\left|\right|R_{j}^{*}||SK_{ij}^{*}\left|\right|T_{4})$. Finally, HG sends the message $\langle p_{i}^{2},M_{G}^{\prime },M_{G,U_{i}},T_{4}\rangle$ to $U_{i}$ over a public channel and updates $PID_{i}^{1}$ stored in its memory to $PID_{i}^{2}$ for $U_{i}$.
(4) Upon receiving the message from HG, $U_{i}$ checks the validity of the timestamp $|T_{4}^{\prime }-T_{4}|<\Delta T$ and computes $R_{j}^{*}=M_{G}^{\prime }⊕h(PID_{i}^{1}||HID_{i}^{*})$, $SK_{ij}^{*}=h(R_{i}\left|\right|R_{j}^{*})$, $C_{i}^{2}=p_{i}^{2}⊕h(HID_{i}^{*}\left|\right|T_{4})$, and $M_{G,U_{i}}^{*}=h(PID_{i}^{1}||HID_{i}^{*}\left|\right|C_{i}^{2}\left|\right|R_{j}^{*}||SK_{ij}^{*}\left|\right|T_{4})$. $U_{i}$ then compares $M_{G,U_{i}}^{*}$ with the received value $M_{G,U_{i}}$. If this condition is not verified, $U_{i}$ terminates this phase since HG fails to prove to be a legitimate home gateway. Otherwise, $U_{i}$ believes that HG is authentic and updates $C_{i}^{1}$ in $SC_{i}$ to $C_{i}^{2}$ for the next session.

### 4.5. Password Change Phase

**Figure 4:**
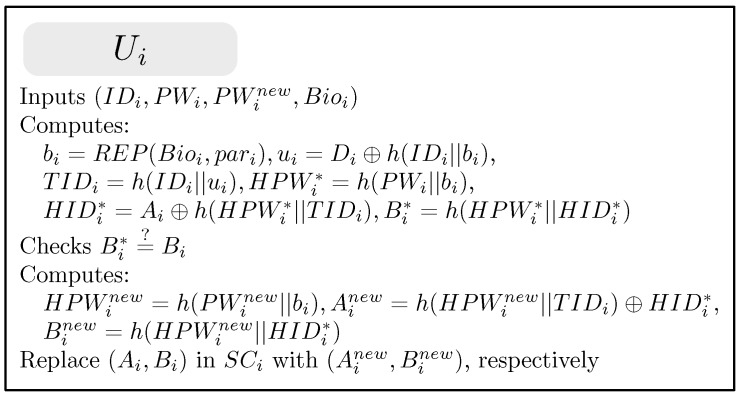
Password change phase for the proposed scheme.

The password change phase begins when $U_{i}$ wants to change the original password $PW_{i}$ to a new password $PW_{i}^{new}$. Figure 4 illustrates this phase for the proposed scheme. This phase contains the following steps.

(1) $U_{i}$ inserts own $SC_{i}$, inputs his/her $ID_{i}$, $PW_{i}$, and a new password $PW_{i}^{new}$ and imprints his/her biometrics $Bio_{i}$ into a terminal.
(2) $SC_{i}$ computes $b_{i}=REP\left(Bio_{i},par_{i}\right)$, $u_{i}=D_{i}⊕H\left(Bio_{i}\right)$, $TID_{i}=h(ID_{i}\left|\right|u_{i})$, $HPW_{i}^{*}=h(PW_{i}\left|\right|b_{i})$, $HID_{i}^{*}=A_{i}⊕h(HPW_{i}^{*}||TID_{i})$, and $B_{i}^{*}=h(HPW_{i}^{*}||HID_{i}^{*})$. $SC_{i}$ then compares $B_{i}^{*}$ with the stored $B_{i}$. If this condition is not satisfied, $SC_{i}$ terminates this phase. Otherwise, $SC_{i}$ performs the next step.
(3) $SC_{i}$ computes $HPW_{i}^{new}=h(PW_{i}^{new}||H\left(Bio_{i}\right))$, $A_{i}^{new}=h(HPW_{i}^{new}||TID_{i})⊕HID_{i}^{*}$, and $B_{i}^{new}=h(HPW_{i}^{new}||HID_{i}^{*})$. $SC_{i}$ replaces the stored values $A_{i}$ and $B_{i}$ with the newly computed values $A_{i}^{new}$ and $B_{i}^{new}$, respectively. Finally, $SC_{i}$ contains $\left\{A_{i}^{new},B_{i}^{new},C_{i}^{\ell },D_{i},h\left(\cdot \right),H\left(\cdot \right)\right\}$, where *ℓ* is the index of the next login.

## 5. Formal Security Analysis of the Proposed Scheme

In this section, we formally analyze the security of the proposed scheme in three ways. First of all, in Section 5.1, we conduct a formal security proof in the random oracle model since the proposed scheme heavily depends on the security of a one-way hash function. Through the rigorous formal poof using the random oracle, we show that the proposed scheme is probabilistically secure against an adversary both to protect the long-term secret information of the user and home gateway and to protect the session key shared between the user and sensor node. In Section 5.2, we then perform the logical verification using BAN logic [26] to confirm the correctness that the authenticated participants share the session key securely in the proposed scheme. In Section 5.3, we automatically validate the proposed scheme using AVISPA tool [27,28] to ensure that the proposed scheme is secure against active and passive attacks (i.e., replay and man-in-the-middle attacks) defined in the simulation tool.

### 5.1. Security Proof Using Random Oracle Model

Through a formal proof using the random oracle model, we show that the proposed scheme is secure against an adversary. We follow the formal security proof of the proposed scheme similar to that in [13,22] and consider the method of contradiction proof. Based on the random oracle model, the following Theorems 1 and 2 show that the proposed scheme can resist various security attacks. For this purpose, we assume that there exists the following random oracle as illustrated in Definition 1.

**Definition** **1.** *Reveal: Given a hash value y=h(x), this random oracle unconditionally outputs the input x.*

**Theorem** **1.** *Under the assumption that a one-way hash function h(·) behaves like an oracle, the proposed scheme is probably secure against an adversary A for deriving the identity $ID_{i}$, the password $PW_{i}$, the biometric key $b_{i}$ of a legal user $U_{i}$ and the secret key $K_{U}$ of the HG, even if the user $U_{i}$’s smart card $SC_{i}$ is lost/stolen.*

**Proof** **of** **Theorem** **1.** For the proof, we assume that an adversary A is able to derive the identity $ID_{i}$, the password $PW_{i}$, and the biometric key $b_{i}$ of a legal user $U_{i}$, and the secret key $K_{U}$ of the HG. We also assume that the adversary A has the lost/stolen smart card $SC_{i}$ of the user $U_{i}$ and A can extract all the sensitive information stored in the $SC_{i}$ using the power analysis attack [29,30,31]. For this, A uses the Reveal oracle to run an experimental algorithm $EXP1_{HASH,A}^{3FAKA}$ shown in Algorithm 1 for the proposed three-factor authentication and key agreement (3FAKA). We define the success probability for $EXP1_{HASH,A}^{3FAKA}$ as $Succ1_{HASH,A}^{3FAKA}=\left|Pr\left[EXP1_{HASH,A}^{3FAKA}=1\right]-1\right|$, where Pr[E] is the probability of an event *E*. The advantage function for this experiment becomes $Adv1_{HASH,A}^{3FAKA}\left(t_{1},q_{R}\right)=max_{A}\left\{Succ1_{HASH,A}^{3FAKA}\right\}$ in which the maximum is taken over all A with execution time $t_{1}$ and the number of queries $q_{R}$ made to the Reveal oracle. According to the attack experiment described in Algorithm 1, if the adversary A has the ability to invert the one-way hash function h(·), then A can directly obtain $U_{i}$’s $ID_{i},PW_{i}$, and $b_{i}$ and HG’s $K_{U}$, and win the game. However, it is computationally infeasible problem to invert h(·), i.e., $Adv1_{HASH,A}^{3FAKA}\left(t_{1}\right)<\epsilon$, for any sufficiently small ϵ>0. Then, we have $Adv1_{HASH,A}^{3FAKA}\left(t_{1},q_{R}\right)\leq \epsilon$, since $Adv1_{HASH,A}^{3FAKA}\left(t_{1},q_{R}\right)\leq \epsilon$ depends on $Adv1_{HASH,A}^{3FAKA}\left(t_{1}\right)$. Therefore, the proposed scheme is provably secure against the adversary A for deriving $ID_{i},PW_{i},b_{i}$, and $K_{U}$, even if the smart card $SC_{i}$ is lost/stolen by A.  □

**Algorithm 1** $EXP1_{HASH,A}^{3FAKA}$
  1:Extract the information $\left\{A_{i},B_{i},C_{i}^{1},D_{i}\right\}$ from $SC_{i}$ using the power analysis attack [29,30,31].  2:Call the Reveal oracle. Let $\left(HPW_{i}^{\prime },HID_{i}^{\prime }\right)\leftarrow Reveal\left(B_{i}\right)$  3:Compute $a=A_{i}⊕HID_{i}^{\prime }$  4:Call the Reveal oracle. Let $\left(HPW_{i}^{\prime \prime },TID_{i}^{\prime }\right)\leftarrow Reveal\left(a\right)$  5:**if** ($HPW_{i}^{\prime \prime }=HPW_{i}^{\prime }$) **then**  6:  Compute $PID_{i}^{1^{\prime }}=C_{i}^{1}⊕h(TID_{i}^{\prime }||HID_{i}^{\prime })$  7:  Intercept the login request message $\langle PID_{i}^{1},M_{i},M_{U_{i},G},T_{1}\rangle$  8:  Call the Reveal oracle. Let $\left(TID_{i}^{*},HID_{i}^{*},PID_{i}^{1*},R_{i}^{*},T_{1}^{*}\right)\leftarrow Reveal\left(M_{U_{i},G}\right)$  9:  **if** ($PID_{i}^{1*}=PID_{i}^{1^{\prime }}$) and ($TID_{i}^{*}=TID_{i}^{\prime }$) and ($HID_{i}^{*}=HID_{i}^{\prime }$) and $\left(T_{1}^{*}=T_{1}\right)$ **then** 10:    Call the Reveal oracle. Let $\left(TID_{i}^{**},K_{U}^{**}\right)\leftarrow Reveal\left(HID_{i}^{\prime }\right)$ 11:    Call the Reveal oracle. Let $\left(PW_{i}^{**},b_{i}^{**}\right)\leftarrow Reveal\left(HPW_{i}^{\prime }\right)$ 12:    Call the Reveal oracle. Let $\left(ID_{i}^{**},u_{i}^{**}\right)\leftarrow Reveal\left(TID_{i}^{\prime }\right)$ 13:    Compute $D_{i}^{\prime }=u_{i}^{**}⊕h(ID_{i}^{**}\left|\right|b_{i}^{**})$ 14:    **if** ($D_{i}^{\prime }=D_{i}$) **then** 15:      Accept $ID_{i}^{**},PW_{i}^{**}$, and $b_{i}^{**}$ as the correct identity $ID_{i}$, password $PW_{i}$, biometric key $b_{i}$ of 16:      the user $U_{i}$, and $K_{U}^{**}$ as the correct secret key $K_{U}$ of HG. 17:      **return** 1 18:    **else** 19:      **return** 0 20:    **end if** 21:  **else** 22:    **return** 0 23:  **end if** 24:
**else**
 25:  **return** 0 26:
**end if**

**Theorem** **2.** *Under the assumption that a one-way hash function h(·) behaves like an oracle, the proposed scheme is probably secure against an adversary A for deriving the session key $SK_{ij}$ shared between a legal user $U_{i}$ and a sensor node $S_{j}$.*

**Proof** **of** **Theorem** **2.** The proof of this theorem is similar to that in Theorem 1. We assume that an adversary A is able to derive the session key $SK_{ij}$ shared between a legal user $U_{i}$ and a sensor node $S_{j}$. For this, A uses the Reveal oracle to run an experimental algorithm $EXP2_{HASH,A}^{3FAKA}$ shown in Algorithm 2 for the proposed three-factor authentication and key agreement (3FAKA). We define the success probability for $EXP2_{HASH,A}^{3FAKA}$ as $Succ2_{HASH,A}^{3FAKA}=\left|Pr\left[EXP2_{HASH,A}^{3FAKA}=1\right]-1\right|$. The advantage function for this experiment becomes $Adv2_{HASH,A}^{3FAKA}\left(t_{2},q_{R}\right)=max_{A}\left\{Succ2_{HASH,A}^{3FAKA}\right\}$ in which the maximum is taken over all A with execution time $t_{2}$ and the number of queries $q_{R}$ made to the Reveal oracle. According to the attack experiment described in Algorithm 2, if the adversary A has the ability to invert the one-way hash function h(·), then A can easily derive $SK_{ij}$ and win the game. However, it is computationally infeasible problem to invert h(·), i.e., $Adv2_{HASH,A}^{3FAKA}\left(t_{2}\right)<\epsilon$, for any sufficiently small ϵ>0. Then, we have $Adv2_{HASH,A}^{3FAKA}\left(t_{2},q_{R}\right)\leq \epsilon$, since $Adv2_{HASH,A}^{3FAKA}\left(t_{2},q_{R}\right)\leq \epsilon$ is also dependent on $Adv2_{HASH,A}^{3FAKA}\left(t_{2}\right)$. Therefore, the proposed scheme is provably secure against the adversary A for deriving $SK_{ij}$.  □

**Algorithm 2** $EXP2_{HASH,A}^{3FAKA}$
  1:Intercept the login request message $\langle PID_{i}^{1},M_{i},M_{U_{i},G},T_{1}\rangle$ during the login phase.  2:Call the Reveal oracle. Let $\left(TID_{i}^{\prime },HID_{i}^{\prime },PID_{i}^{1^{\prime }},R_{i}^{\prime },T_{1}^{\prime }\right)\leftarrow Reveal\left(M_{U_{i},G}\right)$  3:**if** ($PID_{i}^{1^{\prime }}=PID_{i}^{1}$) and ($T_{1}^{\prime }=T_{1}$) **then**  4:  Compute $r_{i}^{\prime }=M_{i}⊕h(TID_{i}^{\prime }||HID_{i}^{\prime }\left|\right|T_{1})$  5:  Compute $R_{i}^{\prime \prime }=h(TID_{i}^{\prime }||PID_{i}^{1}\left|\right|r_{i}^{\prime })$  6:  **if** ($R_{i}^{\prime \prime }=R_{i}^{\prime }$) **then**  7:    Intercept the message $\langle M_{j},M_{S_{j},G},T_{3}\rangle$ during the authentication phase.  8:    Call the Reveal oracle. Let $\left(PID_{i}^{1*},SID_{j}^{*},X_{S_{j}}^{*},R_{j}^{*},SK_{ij}^{*},T_{3}^{*}\right)\leftarrow Reveal\left(M_{S_{j},G}\right)$  9:    **if** ($PID_{i}^{1*}=PID_{i}^{1}$) and ($T_{3}^{*}=T_{3}$) **then** 10:      Compute $SK_{ij}^{\prime }=h(R_{i}^{\prime }\left|\right|R_{j}^{*})$ 11:      **if** ($SK_{ij}^{\prime }=SK_{ij}^{*}$) **then** 12:        Accept $SK_{ij}^{*}$ as the correct session key shared between $U_{i}$ and $S_{j}$. 13:        **return** 1 14:      **else** 15:        **return** 0 16:      **end if** 17:    **else** 18:      **return** 0 19:    **end if** 20:  **else** 21:    **return** 0 22:  **end if** 23:
**else**
 24:  **return** 0 25:
**end if**



### 5.2. Security Verification using BAN Logic

In this section, we use BAN logic to verify the legitimacy of the session key shared between participants who communicate in the proposed scheme. Table 3 and Table 4 illustrate notations and rules used in BAN logic, respectively.

To ensure the security of the proposed scheme under BAN logic, the proposed scheme needs to satisfy the following goals.

- Goal 1: $U_{i}\left|\equiv S_{j}\right|\equiv \left(U_{i}\overset{SK_{ij}}{⟷}S_{j}\right)$
- Goal 2: $U_{i}|\equiv \left(U_{i}\overset{SK_{ij}}{⟷}S_{j}\right)$
- Goal 3: $S_{j}\left|\equiv U_{i}\right|\equiv \left(U_{i}\overset{SK_{ij}}{⟷}S_{j}\right)$
- Goal 4: $S_{j}|\equiv \left(U_{i}\overset{SK_{ij}}{⟷}S_{j}\right)$

We first transfer all transmitted messages into idealized form as follows.

- $M_{1}$: $U_{i}\to HG$: ${\left(PID_{i}^{\ell },R_{i},K_{U},T_{1}\right)}_{HID_{i}}$
- $M_{2}$: $HG\to S_{j}$: ${\left(PID_{i}^{\ell },SID_{j},R_{i},K_{S},T_{2}\right)}_{X_{S_{j}}}$
- $M_{3}$: $S_{j}\to HG$: ${\left(PID_{i}^{\ell },SID_{j},R_{j},K_{S},T_{3}\right)}_{X_{S_{j}}}$
- $M_{4}$: $HG\to U_{i}$: ${\left(PID_{i}^{\ell },PID_{i}^{\ell +1},R_{j},K_{U},T_{4}\right)}_{HID_{i}}$

We secondly define some assumptions as initiative premises as follows.

- $P_{1}$: $HG|\equiv \#(T_{1})$
- $P_{2}$: $S_{j}|\equiv \#\left(T_{2}\right)$
- $P_{3}$: $HG|\equiv \#(T_{3})$
- $P_{4}$: $U_{i}|\equiv \#\left(T_{4}\right)$
- $P_{5}$: $U_{i}|\equiv \left(U_{i}\overset{HID_{i}}{⟷}HG\right)$
- $P_{6}$: $HG|\equiv (U_{i}\overset{HID_{i}}{⟷}HG)$
- $P_{7}$: $S_{j}|\equiv \left(S_{j}\overset{X_{S_{j}}}{⟷}HG\right)$
- $P_{8}$: $HG|\equiv (S_{j}\overset{X_{S_{j}}}{⟷}HG)$
- $P_{9}$: $U_{i}\left|\equiv S_{j}\right|\equiv \left(U_{i}\overset{SK_{ij}}{⟷}S_{j}\right)$
- $P_{10}$: $S_{j}\left|\equiv U_{i}\right|\equiv \left(U_{i}\overset{SK_{ij}}{⟷}S_{j}\right)$

We then prove the proposed scheme achieves the security goals based on the idealized form of the messages, assumptions, and BAN logic rules.

- According to $M_{1}$, we get
  $V_{1}$: $HG◃{\left(PID_{i}^{\ell },R_{i},K_{U},T_{1}\right)}_{HID_{i}}$
- According to $P_{6}$ and Rule 1, we get
  $V_{2}$: $HG|\equiv U_{i}|∼{\left(PID_{i}^{\ell },R_{i},K_{U},T_{1}\right)}_{HID_{i}}$
- According to $P_{1}$ and Rule 3, we get
  $V_{3}$: $HG|\equiv \#{\left(PID_{i}^{\ell },R_{i},K_{U},T_{1}\right)}_{HID_{i}}$
- According to $V_{2},V_{3}$, and Rule 2, we get
  $V_{4}$: $HG|\equiv U_{i}|\equiv {\left(PID_{i}^{\ell },R_{i},K_{U},T_{1}\right)}_{HID_{i}}$
- According to $M_{2}$, we get
  $V_{5}$: $S_{j}◃{\left(PID_{i}^{\ell },SID_{j},R_{i},K_{S},T_{2}\right)}_{X_{S_{j}}}$
- According to $P_{7}$ and Rule 1, we get
  $V_{6}$: $S_{j}\left|\equiv HG\right|∼{\left(PID_{i}^{\ell },SID_{j},R_{i},K_{S},T_{2}\right)}_{X_{S_{j}}}$
- According to $P_{2}$ and Rule 3, we get
  $V_{7}$: $S_{j}|\equiv \#{\left(PID_{i}^{\ell },SID_{j},R_{i},K_{S},T_{2}\right)}_{X_{S_{j}}}$
- According to $V_{6},V_{7}$, and Rule 2, we get
  $V_{8}$: $S_{j}\left|\equiv HG\right|\equiv {\left(PID_{i}^{\ell },SID_{j},R_{i},K_{S},T_{2}\right)}_{X_{S_{j}}}$
- According to $M_{3}$, we get
  $V_{9}$: $HG◃{\left(PID_{i}^{\ell },SID_{j},R_{j},K_{S},T_{3}\right)}_{X_{S_{j}}}$
- According to $P_{8}$ and Rule 1, we get
  $V_{10}$: $HG|\equiv |∼{\left(PID_{i}^{\ell },SID_{j},R_{j},K_{S},T_{3}\right)}_{X_{S_{j}}}$
- According to $P_{3}$ and Rule 3, we get
  $V_{11}$: $HG|\equiv \#{\left(PID_{i}^{\ell },SID_{j},R_{j},K_{S},T_{3}\right)}_{X_{S_{j}}}$
- According to $V_{10},V_{11}$, and Rule 2, we get
  $V_{12}$: $HG|\equiv S_{j}|\equiv {\left(PID_{i}^{\ell },SID_{j},R_{j},K_{S},T_{3}\right)}_{X_{S_{j}}}$
- According to $M_{4}$, we get
  $V_{13}$: $U_{i}◃{\left(PID_{i}^{\ell },PID_{i}^{\ell +1},R_{j},K_{U},T_{4}\right)}_{HID_{i}}$
- According to $P_{5}$ and Rule 1, we get
  $V_{14}$: $U_{i}\left|\equiv HG\right|∼{\left(PID_{i}^{\ell },PID_{i}^{\ell +1},R_{j},K_{U},T_{4}\right)}_{HID_{i}}$
- According to $P_{4}$ and Rule 3, we get
  $V_{15}$: $U_{i}|\equiv \#{\left(PID_{i}^{\ell },PID_{i}^{\ell +1},R_{j},K_{U},T_{4}\right)}_{HID_{i}}$
- According to $V_{14},V_{15}$, and Rule 2, we get
  $V_{16}$: $U_{i}\left|\equiv HG\right|\equiv {\left(PID_{i}^{\ell },PID_{i}^{\ell +1},R_{j},K_{U},T_{4}\right)}_{HID_{i}}$
- As $SK_{ij}=h(R_{i}\left|\right|R_{j})$ and combining $V_{12},V_{16}$, we get
  $V_{17}$: $U_{i}\left|\equiv S_{j}\right|\equiv \left(U_{i}\overset{SK_{ij}}{⟷}S_{j}\right)$ (Goal 1)
- $SK_{ij}=h(R_{i}\left|\right|R_{j})$ and combining $V_{4},V_{8}$, we get
  $V_{18}$: $S_{j}\left|\equiv U_{i}\right|\equiv \left(U_{i}\overset{SK_{ij}}{⟷}S_{j}\right)$ (Goal 3)
- According to $P_{9},V_{17}$ and Rule 4, we get
  $V_{19}$: $U_{i}|\equiv \left(U_{i}\overset{SK_{ij}}{⟷}S_{j}\right)$ (Goal 2)
- According to $P_{10},V_{18}$ and Rule 4, we get
  $V_{20}$: $S_{j}|\equiv \left(U_{i}\overset{SK_{ij}}{⟷}S_{j}\right)$

Therefore, the above logic proves that the proposed scheme achieves Goals 1–4 successfully. In other words, the proposed scheme achieves mutual authentication and the session key $SK_{ij}$ is securely shared between parties.

### 5.3. Security Verification Using AVISPA

We simulate the proposed scheme using the AVISPA software, a widely accepted tool for automatically validating the security features of the protocols. We describe the implementation of the proposed scheme using HLPSL (High-Level Protocols Specification Language) and then present the simulation results.

#### 5.3.1. HLPSL Specification of the Proposed Scheme

We now briefly discuss the simulation process of the proposed scheme for the roles of the participants, $U_{i},HG$, and $S_{j}$, the session, the goal, and the environment. Table 5, Table 6 and Table 7 present the roles of $U_{i},HG$, and $S_{j}$ in HLPSL language, respectively. Table 8 presents the session, environment, and goal roles in the HLPSL language. In the implementation, the following seven secrecy goals and two authentication properties were verified.

- Goal 1: The secrecy_of subs1 represents that $\langle ID_{i},PW_{i}\rangle$ are kept secret to ($U_{i}$) only.
- Goal 2: The secrecy_of subs2 represents that $\langle TID_{i},HID_{i}\rangle$ are kept secret to ($U_{i},HG$) only.
- Goal 3: The secrecy_of subs3 represents that $\langle R_{i},R_{j}\rangle$ are kept secret to ($U_{i},HG,S_{j}$) only.
- Goal 4: The secrecy_of subs4 represents the negotiated session key $SK_{ij}$ is only known to ($U_{i},HG,S_{j}$).
- Goal 5: The secrecy_of subs5 represents that the secret key $K_{U}$ of HG is permanently kept secret, known to only (HG).
- Goal 6: The secrecy_of subs6 represents that the secret key $K_{S}$ of HG is permanently kept secret, known to only (HG).
- Goal 7: The secrecy_of subs7 represents that the shared secret $X_{S_{j}}$ is only known to ($HG,S_{j}$).
- Authentication Property 1: The authentication_on user_gateway_rri represents that $U_{i}$ generates $R_{i}$. If HG securely receives $R_{i}$ through a message, it authenticates $U_{i}$.
- Authentication Property 2: The authentication_on gateway_sensor_rrj represents that $S_{j}$ generates $R_{j}$. If HG securely receives $R_{j}$ through a message, it authenticates $S_{j}$.

#### 5.3.2. Simulation Results

**Figure 5:**
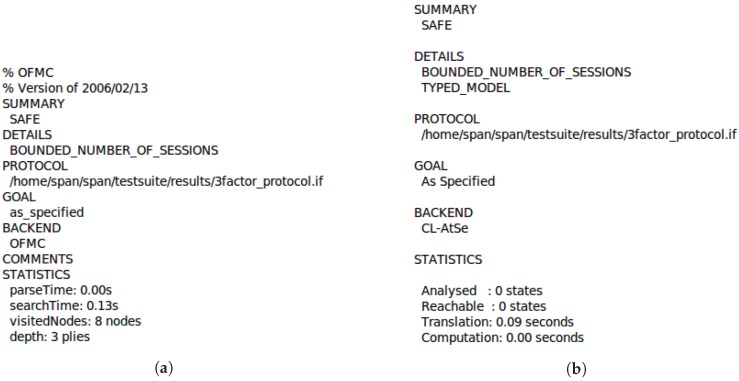
Simulation results of the proposed scheme using AVISPA tool: (**a**) OFMC model, (**b**) CL-AtSe model.

We execute the HLPSL specifications using SPAN (Security Protocol ANimator for AVISPA) [32]. Figure 5a,b show the simulation results based on OFMC (On-the-Fly-Model-Checker) and CL-AtSe (Constraint-Logic-based Attack Searcher) models, respectively. From these results, we find that the proposed scheme is SAFE under OFMC and CL-AtSe against active and passive attacks. Therefore, we demonstrate that the proposed scheme is secure.

## 6. Implication of Security Analysis

We further describe the implication of our security analysis with regard to security properties of the proposed scheme. Saying, we show how the proposed scheme satisfies the security requirements for user authentication and session key agreement and resists various kinds of known attacks. We then compare the security of the proposed scheme with other related schemes.

### 6.1. Security Properties

#### 6.1.1. Mutual Authentication

In steps (1) and (4) of Section 4.4, $U_{i}$ and HG authenticate each other by verifying the correctness of $M_{U_{i},G}$ and $M_{G,U_{i}}$. An adversary cannot generate legal $M_{U_{i},G}=h(TID_{i}||HID_{i}^{*}||PID_{i}^{1}|R_{i}\left|\right|T_{1})$ and $M_{G,U_{i}}=h(PID_{i}^{1}||HID_{i}^{*}\left|\right|C_{i}^{2}\left|\right|R_{j}^{*}||SK_{ij}^{*}\left|\right|T_{4})$ without knowing $HID_{i}$. Even if the adversary obtains $SC_{i}$ of $U_{i}$ and stored values, the adversary cannot derive the correct $HID_{i}$ without having the corresponding $U_{i}$’s $ID_{i},PW_{i}$, and $Bio_{i}$. As a result, the proposed scheme can achieve mutual authentication between $U_{i}$ and HG.

In steps (2) and (3) of Section 4.4, HG and $S_{j}$ authenticate each other by verifying the correctness of $M_{G,S_{j}}$ and $M_{S_{j},G}$. An adversary cannot generate legal $M_{G,S_{j}}=h(PID_{i}^{1}||SID_{j}\left|\right|X_{S_{j}}\left|\right|R_{i}^{*}\left|\right|T_{2})$ and $M_{S_{j},G}=h(PID_{i}^{1}||SID_{j}\left|\right|X_{S_{j}}\left|\right|R_{j}||SK_{ij}\left|\right|T_{3})$ without knowing their shared secret information $X_{S_{j}}$. As a result, the proposed scheme can achieve mutual authentication between HG and $S_{j}$.

#### 6.1.2. Session Key Agreement

In the login and authentication phases, the session key $SK_{ij}=h(R_{i}\left|\right|R_{j})=h(h(TID_{i}||PID_{i}^{\ell }\left|\right|r_{i})||h(SID_{j}\left|\right|r_{j}))$ is established between $U_{i}$ and $S_{j}$ for protecting future communication. In the proposed scheme, the secrecy of $SK_{ij}$ is dependent on the secrecy of the random values $r_{i}$ and $r_{j}$. These values are carefully protected by the secret keys shared between $U_{i}$ and HG and between HG and $S_{j}$, respectively. Even if an adversary obtains $SK_{ij}$ for the *ℓ*-th session, he/she cannot compute any of the past and future session keys by using this disclosed $SK_{ij}$ because $SK_{ij}$ is protected by h(·) and the random values $r_{i}$ and $r_{j}$ including one-time psuedonym $PID_{i}^{\ell }$ are different in each session. As a result, the proposed scheme achieves both session key agreement and known key security.

#### 6.1.3. User Anonymity with Untraceability

As we mentioned in Section 3.1, for fully protecting user privacy, strong anonymity with untraceability is required. In the proposed scheme, the $U_{i}$’s actual identity $ID_{i}$ is not transmitted during all phases, including the registration phase. Therefore, even if an adversary eavesdrops on all communication messages, it is not possible to obtain $ID_{i}$ directly from the messages. In addition, even if the adversary gets $TID_{i}$, it cannot retrieve $ID_{i}$ from $TID_{i}$ because $ID_{i}$ is masked with $u_{i}$ and $u_{i}$ is protected by $Bio_{i}$ only known to $U_{i}$. Similarly, even if the adversary gets $HID_{i}$, it cannot retrieve $ID_{i}$ from $HID_{i}$ without knowing a secret key, $K_{U}$, which is only known to HG.

Furthermore, $M_{i}$ and $M_{U_{i},G}$ in the login request message are computed with random values $r_{i}$ and $T_{1}$ and $U_{i}$ uses an one-time pseudonym $PID_{i}^{\ell }$ every session. In other words, all values in the login request message are different in sessions. Therefore, any adversary cannot trace the different sessions of the same user from exchanged messages via public channels and the proposed scheme achieves the feature of strong anonymity with untraceability.

#### 6.1.4. Resisting Stolen Smart Card Attack

In the proposed scheme, $U_{i}$’s smart card $SC_{i}$ contains $\left\{A_{i},B_{i},C_{i}^{\ell },D_{i},par_{i},h\left(\cdot \right),GEN\left(\cdot \right),REP\left(\cdot \right)\right\}$ where $A_{i}=h(HPW_{i}||TID_{i})⊕HID_{i}$, $B_{i}=h(HPW_{i}||HID_{i})$, $C_{i}^{\ell }=h(TID_{i}||HID_{i})⊕PID_{i}^{\ell }$ and $D_{i}=u_{i}⊕h(ID_{i}\left|\right|b_{i})$. Even if $SC_{i}$ is stolen by an adversary and all contained values in it are retrieved by the adversary through side-channel attacks such as power analysis attack [29,30,31], the adversary cannot guess $HPW_{i}$, $TID_{i}$, and $HID_{i}$ including $ID_{i}$, $PW_{i}$, and $Bio_{i}$ by using $A_{i}$, $B_{i}$, $C_{i}$, and $D_{i}$ and also cannot guess $PID_{i}^{\ell }$ from $C^{\ell }$ without knowing $b_{i},u_{i}$, and $K_{U}$ because it is impossible to know these key values. Without knowing $U_{i}$’s real identity $ID_{i}$, password $PW_{i}$, and biometric $Bio_{i}$, the adversary cannot impersonate as the user. As a result, the proposed scheme can resist the stolen smart card attack.

#### 6.1.5. Resisting Offline Guessing Attack

An adversary may attempt to guess $U_{i}$’s identity $ID_{i}$, password $PW_{i}$ and biometric key $b_{i}$ by extracting the values stored in the smart card $SC_{i}$. However, the adversary cannot derive $b_{i}$ using only $par_{i}$ without knowing the $U_{i}$’s biometric $Bio_{i}$. The adversary also cannot derive $ID_{i}$ and $b_{i}$ from $TID_{i}$ and $D_{i}$, respectively, without knowing the random value $u_{i}$. Therefore, the adversary cannot guess the correct $ID_{i}$, $PW_{i}$, and $b_{i}$ without knowing $Bio_{i}$ and $u_{i}$ due to the collision-resistant property of the one-way hash function h(·). As a result, the proposed scheme can resist the offline guessing attack.

#### 6.1.6. Resisting Privileged Insider Attack

In practice, users tend to use same password to register across different systems. If a privileged insider obtain the user’s password, he/she can use it to access other systems by impersonating as this user. In the proposed scheme, $U_{i}$ submits the hashed password $HPW_{i}$ instead of the plaintext of real password $PW_{i}$ during the registration phase. $HPW_{i}$ is also masked by $U_{i}$’s secret biometric key $b_{i}$. Therefore, an insider cannot obtain $U_{i}$’s real password and the proposed scheme can resist the privileged insider attack.

#### 6.1.7. Resisting Stolen-Verifier Attack

To succeed in the stolen-verifier attack, an adversary should obtain the verification information (e.g., the plaintexts of passwords, hashed passwords, biometric key data, or hashed biometric key data) stored in the server. However, in the proposed scheme, the server maintains only $\left\{PID_{i}^{1},TID_{i}\right\}$ which is both password-independent and biometric-key-independent information. Therefore, the proposed scheme can resist the stolen-verifier attack.

#### 6.1.8. Resisting Known Session-Specific Temporary Information Attack

In the proposed scheme, both randomly selected values $r_{i}$ and $r_{j}$, from $U_{i}$ and $S_{j}$, respectively, are always masked by the secret values $HID_{i}$ and $X_{S_{j}}$. Even if an adversary knows $r_{i}$ and $r_{j}$, he/she cannot compute $SK_{ij}=h(R_{i}\left|\right|R_{j})=h(h(TID_{i}||PID_{i}^{\ell }\left|\right|r_{i})||h(SID_{j}\left|\right|r_{j}))$ without knowing $U_{i}$’s temporary identity $TID_{i}$ and one-time pseudonym $PID_{i}^{\ell }$ and $S_{j}$’s identity $SID_{j}$. Moreover, as we described, the adversary has no way to compute $TID_{i}$ and $SID_{j}$. As a result, in the proposed scheme, a leakage of the session-specific temporary information $r_{i}$ and $r_{j}$ does not affect the security of the established session key.

#### 6.1.9. Resisting User Impersonation Attack

To impersonate a user $U_{i}$, an adversary should obtain the values in $SC_{i}$ and intercepts the messages exchanged in the previous sessions. In the proposed scheme, even if the adversary succeeded the above things, the adversary cannot produce a legal login request $\langle PID_{i}^{1},M_{i},M_{U_{i},G},T_{1}\rangle$ without knowing all the authentication factors, i.e., $SC_{i}$, $PW_{i}$, and $Bio_{i}$ including $ID_{i}$ and $u_{i}$. As we mentioned above, it is impossible for an adversary to obtain $ID_{i},PW_{i},u_{i}$, and $b_{i}$. Therefore, the proposed scheme can resist the user impersonation attack.

#### 6.1.10. Resisting Sensor Node Impersonation and Node Capture Attacks

To impersonate a sensor node $S_{j}$, an adversary should intercept the messages exchanged in the previous sessions. However, in the proposed scheme, the adversary cannot produce a legal message $\langle M_{j},M_{S_{j},G},T_{3}\rangle$ without knowing $X_{S_{j}}=h(SID_{j}\left|\right|K_{S})$ because the adversary does not know the HG’s secret key $K_{S}$ even if he/she obtains $SID_{j}$.

Even if the adversary captures a sensor node $S_{j}$ and obtains $X_{S_{j}}$ stored in $S_{j}$, the adversary’s further attacks using the compromised sensor node only affect communications related to that node. Since each sensor node has a different key $X_{S_{m}}=h(SID_{m}\left|\right|K_{S})$, the adversary cannot derive other non-compromised sensor nodes’ keys without knowing $K_{S}$ and thus the further attacks will not affect other communications. As a result, the proposed scheme can resist both sensor node impersonation attack and node capture attack.

### 6.2. Comparison of Security Features

We compare the security features of the proposed scheme with other related three-factor authentication and key agreement schemes [9,11,12,13]. Table 9 shows the comparison results. From Table 9, we can see that first three related schemes do not guarantee all security features, in especial, untraceability required for strong anonymity. The proposed scheme and Jiang et al.’s scheme achieves more ideal security features and resist most of attacks. However, Jiang et al.’s scheme is expensive to implement and deploy in practical applications due to the low performance of Rabin cryptosystem. As shown in Section 7, Jiang et al.’s scheme is five times slower than the proposed scheme in total running time.

## 7. Performance Analysis of the Proposed Scheme

We analyze the performance of the proposed scheme and compare it with other related schemes in terms of computational cost and communication cost.

### 7.1. Computational Cost Analysis

For computational cost analysis, we compare the computation cost of the proposed scheme with the four related schemes [9,11,12,13]. We only focus on comparing the login and authentication phases because the registration and password change phases are not performed frequently. Since the time for executing of a bitwise XOR operation is negligible, we do not consider XOR operations for computational cost analysis. To facilitate analysis, we use the following notations.

- $T_{H}$: time for executing a one-way hash function
- $T_{B}$: time for executing a biohash function
- $T_{F}$: time for executing a fuzzy extractor
- $T_{P}$: time for executing an ECC point multiplication
- $T_{M}$: time for a modular exponentiation

Wang et al. [33] implemented several operations on three kinds of common PCs and measured their execution time by using C/C++ library MIRACL. According to the experimental results in Wang et al.’s research [33], we assume that the executing time for the cryptographic one-way hash function $T_{H}$ (SHA-1), ECC point multiplication $T_{P}$ (ECC sect163r1 [34]), and modular exponentiation $T_{M}$ (|n|=512) on common PCs (Intel T5870 2.00 GHz, Intel, Santa Clara, CA, US) are 2.58 µs, 1.226 ms, and 2.573 ms, respectively. Moreover, the execution time for the fuzzy extractor operation $T_{F}$ is almost the same as the ECC point multiplication $T_{P}$ [35] and it is also assumed that $T_{B}=T_{F}\approx T_{P}$ according to [36]. We consider possible real sensor devices with 8-bit ATmega128L micorocontroller (i.e., MICAz of Crossbow Technology). According to the experimental results on those sensor nodes [37,38], we assume that the executing time for the cryptographic one-way hash function $T_{H}^{\prime }$ (SHA-1) and ECC point multiplication $T_{P}^{\prime }$ (ECC sect163r1 [34]) are 3.6 ms and 114 ms, respectively.

In Table 10, we summarize the computational cost and running time of the proposed scheme and of the related schemes for user, gateway node, and sensor node. The total running time of the proposed scheme for the login and authentication phases is $T_{F}+28T_{H}+6T_{H}^{\prime }\approx 22.9$ ms. It shows that the proposed scheme is almost 10 times more efficient than and Park et al. scheme [9]. The proposed scheme also has a higher security level than both Amin et al.’s scheme [11] and Jung et al.’s scheme [13] as shown in Table 9 and it is as efficient as them. Although Jiang et al.’s scheme [12] has similar security level with the proposed scheme, the proposed scheme is slightly efficient and easily implemented than Jiang’s et al.’s scheme since the proposed scheme uses only lightweight operations such as XOR and hash functions not complex public-key cryptographic operations. Therefore, the proposed scheme can achieve all security features in Table 9 without deteriorating efficiency in terms of the computational cost.

### 7.2. Communication Cost Analysis

We also analyze the communication cost of the proposed scheme for login and authentication phases and compare it with that of the related schemes [9,11,12,13]. For communication cost analysis, we evaluate the communication cost in terms of the size of message in bits and the number of values in a message. We assume that the lengths of the identity, password, random number, and output of the hash function are each 128 bits. We also assume that the lengths of modulo *n* for rabin cryptosystem used in [12] and prime *p* for ECC used in [9] are each 1024 bits.

The communication cost of user, gateway node, and sensor node of the proposed scheme and related schemes are summarized in Table 11. The total communication cost of the proposed scheme is 1920 bits. From comparison in Table 11, the proposed scheme require lower communication cost than the above related schemes expect Jung et al.’s scheme. Although the proposed scheme is slightly less efficient than Jung et al.’s scheme in terms of communication cost, the difference (512 bits) is not significant since the proposed scheme has a higher security level as shown in Table 9.

## 8. Conclusions

In this paper, we have identified the security weaknesses in the recent three-factor authentication and key agreement scheme. Then, we have introduced the system model for smart homes based on WSNs. Based on this model, we have proposed a secure and lightweight three-factor authentication and key agreement scheme using the smart card, password, and biometrics. We have presented security proof using random oracle model and BAN logic. Afterwards, we have performed the security verification using AVISPA. Through formal and informal security analysis, we have demonstrated the proposed scheme fulfills the desirable security requirements and resists against various attacks. We have also evaluated the performance of the proposed scheme with regard to the computational and communication overheads. Finally, we have presented the comparative analysis of the proposed scheme with other related schemes, which justify that the proposed scheme has advantages in terms of efficiency and security.

In the future work, we expect to evaluate the performance of the proposed scheme by implementing and conducting experiments on actual devices (e.g., smart phones and sensor motes) for smart homes based on WSNs. Based on the experimental results, it will be possible to further examine the effectiveness of the proposed scheme.

## Figures and Tables

**Table 1:** Notations for Jung et al.’s scheme.

Notation	Description	Notation	Description
$U_{i}$	Remote user	*u*	Random number of $U_{i}$
$S_{j}$	Sensor node	*R*	Random number
GWN	Gateway node	*K*	Secret key generated by the GWN
$ID_{i},PW_{i}$	Identity and password of $U_{i}$	$K_{S}$	Session key
$Bio_{i}$	Biometric information of $U_{i}$	f(v,k)	Pseudo-random function of variable *v* with key *k*
$TID_{i}$	Temporary identity of $U_{i}$’s next login	h(·),H(·)	One-way hash function and biohash function
$SID_{j}$	Identity of $S_{j}$	T,ΔT	Timestamp and the transmission delay time

**Table 2:** Notations used for the proposed scheme.

Notation	Description	Notation	Description
HG	Home Gateway	$SC_{i}$	Smart card for $U_{i}$
$TID_{i}$	Temporary identity of $U_{i}$	SK	Session key
$PID_{i}^{l}$	One-time pseudonym of $U_{i}$ for the *l*-th login	GEN(·)	Fuzzy generator function
$K_{U}$	Secret key generated by the HG for users	REP(·)	Fuzzy reproduction function
$K_{S}$	Secret key generated by the HG for sensor nodes		

**Table 3:** Notations in BAN logic.

Notation	Description	Notation	Description
P|≡X	*P* believes *X*	#(X)	*X* is fresh
P◃X	*P* sees *X*	$P\overset{K}{↔}Q$	*K* is the shared key between *P* and *Q*
P|∼X	*P* said *X*	${\langle X\rangle }_{Y}$	*X* combined with the formula *Y*
P⇒X	*P* has jurisdiction over *X*	${\left(X\right)}_{K}$	*X* hashed under the key *K*

**Table 4:** Rules in BAN logic.

Rule	Description
$\frac{P|\equiv P\overset{K}{↔}Q,P◃{\langle X\rangle }_{K}}{P|\equiv Q|∼X}$	**[Rule 1: Message-meaning rule]** if *P* believes that the *K* is shared with *Q* and *P* sees *X* combined with *K*, then *P* believes *Q* said *X*
$\frac{P|\equiv \#(X),P|\equiv Q|∼X}{P|\equiv Q|\equiv X}$	**[Rule 2: Nonce-verification rule]** if *P* believes that *X* is fresh and *P* believes *Q* said *X*, then *P* believes that *Q* believes *X*
$\frac{P|\equiv \#(X)}{P|\equiv \#(X,Y)}$	**[Rule 3: Freshness-conjuncation rule]** if *P* believes that *X* is fresh, then *P* believes that (X,Y) is fresh
$\frac{P|\equiv Q|\Rightarrow X,P|\equiv Q|\equiv X}{P|\equiv X}$	**[Rule 4: Jurisdiction rule]** if *P* believes that *X* has jurisdiction over *X* and *P* believes that *Q* believes *X*, then *P* also believes *X*

**Table 5:** Role specification of $U_{i}$ in HLPSL.

1: role user(Ui, HG, Sj: agent, SKey1: symmetric_key, SKey2: symmetric_key,
H, GEN, REP: hash_func, Snd, Rcv: channel(dy))
2: played_by Ui
3: def=
4: local State: nat, IDi, PWi, Bioi, BBi, Pari, TIDi, HPWi, HIDi, PID1i, Si, Ai, Bi, Ci, C1i, C2i, Di, RRi, RRj, Ri,
T1, T4: text, Mi, Muig, SKij, P2i, Mg, Mgui: message,
5: Inc: hash_func
6: const user_gateway, gateway_user, sensor_user, subs1, subs2, subs3, subs4, subs5, subs6, subs7: protocol_id
7: init State :=0
8: transition
9: 1. State = 0 ∧ Rcv(start) =|>
10: State’ := 1 ∧ IDi’ := new() ∧ PWi’ := new() ∧ Si’ := new() ∧ BBi’ := GEN(Bioi) ∧ Pari’ := GEN(Bioi)
∧ HPWi’ := H(PWi’.BBi’) ∧ TIDi’ := H(IDi’.Si’) ∧ Snd(TIDi’.HPWi’_SKey1) ∧ secret(IDi,PWi, subs1, Ui)
11: 2. State = 1 ∧ Rcv({Ai’.Bi’.C1i’}_SKey1) =|>
12: State’ := 2 ∧ Ri’ := new() ∧ T1’ := new() ∧ BBi’ := GEN(Bioi) ∧ Di’ := xor(ui,H(IDi.BBi’)) ∧ TIDi’ := H(IDi.ui)
∧ HPWi’ := h(PWi.BBi’) ∧ Ai’ := xor(HIDi, H(HPWi’.TIDi’)) ∧ Bi’ := H(HPWi’.HIDi)
∧ PID1i’ := xor(C1i’, H(TIDi’.HIDi)) ∧ RRi’ := H(TIDi’.PID1i’.Ri’) ∧ Mi’ := xor(Ri’, H(TIDi’.HIDi.T1’))
∧ Muig’ := H(TIDi’.HIDi.PID1i’.RRi’.T1’)
∧ Snd(PID1i’.Mi’.Muig’.T1’) ∧ secret({TIDi,HIDi}, subs2, {Ui, HG}) ∧ witness(Ui, HG, user_gateway, RRi’)
13: 3. State = 2 ∧ Rcv(P2i’.Mg’.Mgui’.T4’) =|>
14: State’ := 3 ∧ RRj’ := xor(Mg’, H(PID1i.HIDi)) ∧ SKij’ := H(RRi.RRj’)
∧ secret({RRi,RRj}, subs3, {Ui, HG, Sj}) ∧ secret({SKij}, subs4, {Ui, HG, Sj})
15: end role

**Table 6:** Role specification of HG in HLPSL.

1: role gateway(Ui, HG, Sj: agent, SKey1: symmetric_key, SKey2: symmetric_key,
H, GEN, REP: hash_func, Snd, Rcv: channel(dy))
2: played_by HG
3: def=
4: local State: nat, Ku, Ks, TIDi, HPWi, PID1i, PID2i, HIDi, Ai, Bi, C1i, C2i, SIDj, Xsj, RRi, RRj, Ri, Rj,
T1, T2, T3, T4: text, Mi, Muig, Mg, Mg2, Mgsj, Mgui, Mj, Msjg, SKij, P2i: message,
5: Inc: hash_fun
6: const user_gateway, gateway_user, sensor_user, subs1, subs2, subs3, subs4, subs5, subs6, subs7: protocol_id
7: init State :=0
8: transition
9: 1. State = 0 ∧ Rcv({TIDi’.HPWi’}_SKey1) =|>
10: State’ := 1 ∧ PID1i’ := new() ∧ HIDi’ := H(TIDi’.Ku) ∧ Ai’ := xor(HIDi’, H(HPWi’.TIDi’))
∧ Bi’ := H(HPWi’.HIDi’) ∧ C1i’ := xor(PID1i’, H(TIDi’.HIDi’)) ∧ Snd({Ai’.Bi’.C1i’}_SKey1)
∧ SIDj’ := new() ∧ Xsj’ := H(SIDj’.Ks)
∧ Snd({SIDj’.Xsj’}_SKey2) ∧ secret(Ku, subs5, HG) ∧ secret(Ks, subs6, HG) ∧ secret(Xsj, subs7, HG, Sj)
11: 2. State = 1 ∧ Rcv(PID1i’.Mi’.Muig’.T1’) =|>
12: State’ := 2 ∧ HIDi’ := H(TIDi.Ku) ∧ Ri’ := xor(Mi’, H(TIDi.HIDi’.T1’)) ∧ RRi’ := H(TIDi.PID1i.Ri’)
∧ T2’ := new() ∧ Xsj’ := H(SIDj.Ks) ∧ Mg2’ := xor(RRi’, H(Xsj’.T2’)) ∧ Mgsj’ := H(PID1i’.SIDj.Xsj’.RRi’.T2’)
∧ Snd(PID1i’.Mg2’.Mgsj’.T2’)
13: 3. State = 2 ∧ Rcv(Mj’.Msjg’.T3’) =|>
14: State’ := 3 ∧ Rj’ := xor(Mj’, H(Xsj.T3’)) ∧ RRj’ := H(SIDj.Rj’) ∧ SKij’ := H(RRi.RRj’) ∧ PID2i’ := new()
∧ T4’ := new() ∧ C2i’ := xor(PID2i’, H(TIDi.HIDi)) ∧ P2i’ := xor(C2i’, H(HIDi.T4’))
∧ Mg’ := xor(RRj’, H(PID1i.HIDi)) ∧ Mgui’ := H(PID1i.HIDi.C2i’.RRj’.SKij’.T4’)
∧ Snd(P2i’.Mg’.Mgui’.T4’)
15: end role

**Table 7:** Role specification of $S_{j}$ in HLPSL.

1: role sensor(Ui, HG, Sj: agent, SKey1: symmetric_key, SKey2: symmetric_key,
H, GEN, REP: hash_func, Snd, Rcv: channel(dy))
2: played_by Sj
3: def=
4: local State: nat, PID1i, SIDj, Xsj, RRi, RRj, Rj, T2, T3: text, Mg2, Mgsj, Mgui, Mj, Msjg, SKij: message,
5: Inc: hash_func
6: const user_gateway, gateway_sensor, sensor_user, subs1, subs2, subs3, subs4, subs5, subs6, subs7: protocol_id
7: init State :=0
8: transition
9: 1. State = 0 ∧ Rcv({SIDj’.Xsj’}_SKey2) =|>
10: State’ := 1 ∧ T3’ := new()
11: 2. State = 1 ∧ Rcv(PID1i’.Mg2’.Mgsj’.T2’) =|>
12: State’ := 2 ∧ RRi’ := xor(Mg2’, H(Xsj.T2’)) ∧ Rj’ := new() ∧ T3’ := new() ∧ RRj’ := H(SIDj.Rj’)
∧ Mj’ := xor(Rj’, H(Xsj.T3’)) ∧ SKij’ := H(RRi’.RRj’) ∧ Msjg’ := H(PID1i’.SIDj.Xsj.Rj’.SKij’.T3’)
∧ Snd(Mj’.Msjg’.T3’) ∧ witness(Sj, HG, gateway_sensor, RRj’)
13: end role

**Table 8:** Specification of the session, environment, and goal in HLPSL.

1: role session(Ui, HG, Sj:agent, SKey1: symmetric_key, SKey2: symmetric_key,
H, GEN, REP: hash_func)
2: def=
3: local SI, SJ, RI, RJ, PI, PJ: channel(dy)
4: composition
5: user(Ui, HG, Sj, SKey1, SKey2, H, GEN, REP, SI, RI)
6: ∧ gateway(Ui, HG, Sj, SKey1, SKey2, H, GEN, REP, SJ, RJ)
7: ∧ sensor(Ui, HG, Sj, SKey1, SKey2, H, GEN, REP, PI, PJ)
8: end role
1: role environment()
2: def=
3: const ui, hg, sj: agent, skey1 : symmetric_key, skey2 : symmetric_key, h, gen, rep: hash_func,
4: idi, bioi, sidj, pwi, ai, bi, ci, t1, t2, t3, t4, rri, rrj, skij, mi, mj, mg, mg2, muig, mgui, mgsj, msjg: text,
5: user_gateway_rri, gateway_sensor_rrj, sensor_user,
6: subs1, subs2, subs3, subs4, subs5, subs6, subs7: protocol_id
7: intruder_knowledge = ui, hg, sj, h, gen, rep, mi, muig, mg2, mgsj, mj, msjg, mg, mgui
8: composition
9: session(hg, ui, sj, skey1, skey2, h, gen, rep)
10: ∧ session(ui, hg, sj, skey1, skey2, h, gen, rep)
11: ∧ session(sj, ui, hg, skey1, skey2, h, gen, rep)
12: end role
1: goal
2: secrecy_of subs1 secrecy_of subs2 secrecy_of subs3 secrecy_of subs4
3: secrecy_of subs5 secrecy_of subs6 secrecy_of subs7
4: authentication_on user_gateway_rri authentication_on gateway_sensor_rrj 5: end goal
environment()

**Table 9:** Security feature comparison of the proposed scheme with other related three-factor authentication and key agreement schemes.

Security Feature	Amin et al. [11]	Park et al. [9]	Jung et al. [13]	Jiang et al. [12]	Proposed Scheme
Mutual authentication	O	O	O	O	O
Session key security	O	O	X	O	O
User anonymity	O	O	O	O	O
Untraceability	X	X	X	O	O
Resistance to					
Stolen smart card attack	X	O	X	O	O
Offline guessing attack	O	O	O	O	O
Privileged insider attack	O	O	O	O	O
Stolen-verifier attack	O	X	O	O	O
Known session-specific	X	O	O	O	O
temporary information attack					
User impersonation attack	O	O	X	O	O
Sensor node	O	O	O	O	O
impersonation attack					

O: The scheme can provide the security feature or resist the attack; X: The scheme cannot provide the security feature or resist the attack.

**Table 10:** Comparison of computation costs for the login and authentication phases of the proposed scheme and other related schemes.

Entity	Amin et al. [11]	Park et al. [9]	Jung et al. [13]	Jiang et al. [12]	Proposed Scheme
User	$T_{B}+12T_{H}$	$T_{F}+2T_{P}+10T_{H}$	$T_{B}+8T_{H}$	$T_{B}+T_{M}+8T_{H}$	$T_{F}+13T_{H}$
Gateway node	$15T_{H}$	$11T_{H}$	$9T_{H}$	$T_{M}+12T_{H}$	$15T_{H}$
Sensor node	$5T_{H}^{\prime }$	$2T_{P}^{\prime }+4T_{H}^{\prime }$	$4T_{H}^{\prime }$	$5T_{H}^{\prime }$	$6T_{H}^{\prime }$
Total cost	$T_{B}+27T_{H}+5T_{H}^{\prime }$	$T_{F}+2T_{P}+2T_{P}^{\prime }$	$T_{B}+17T_{H}+4T_{H}^{\prime }$	$T_{B}+2T_{M}$	$T_{F}+28T_{H}+6T_{H}^{\prime }$
		$+21T_{H}+4T_{H}^{\prime }$		$+20T_{H}+5T_{H}^{\prime }$	
Total running time	≈19.3 ms	≈246.1 ms	≈15.7 ms	≈24.4 ms	≈22.9 ms

**Table 11:** Comparison of communication costs for the login and authentication phases of the proposed scheme and other related schemes: the size of message in bits (the number of values in a message).

Communication	Amin et al. [11]	Park et al. [9]	Jung et al. [13]	Jiang et al. [12]	Proposed Scheme
User→Gateway node	768 bits (6)	1536 bits (5)	512 bits (4)	1408 bits (4)	512 bits (4)
Gateway node→Sensor node	640 bits (5)	1408 bits (4)	512 bits (4)	640 bits (5)	512 bits (4)
Sensor node→Gateway node	384 bits (3)	1280 bits (3)	256 bits (2)	384 bits (3)	384 bits (3)
Gateway node→User	384 bits (3)	1408 bits (4)	384 bits (3)	256 bits (2)	512 bits (4)
Total	2176 bits	5632 bits	1664 bits	2688 bits	1920 bits
